# A novel low-cost model of superficial abscess for trainee education in incision and drainage

**DOI:** 10.1016/j.sopen.2023.07.015

**Published:** 2023-07-27

**Authors:** Joseph M. Ladowski, Lillian Kang, Layla Triplett, Brian Fogler, John Migaly, Sabino Zani, Louise Jackson, Cory J. Vatsaas

**Affiliations:** a Duke University, Department of Surgery, Duke University School of Medicine, Durham, NC 27710, USA; b Duke University, Department of Radiology, Duke University School of Medicine, Durham, NC 27710, USA

**Keywords:** Surgical simulation, Superficial abscess, Incision and drainage, Ultrasound

## Abstract

**Background:**

Proficiency in ultrasound usage is quickly becoming an expectation in multiple residency programs: emergency medicine, obstetrics-gynecology, surgery, and internal medicine. There is a lack of affordable training devices for ultrasound training and identification of superficial fluid collections. We sought to develop a model for trainee education in ultrasound usage, identification of superficial fluid collection, aspiration, and incision & drainage (I&D).

**Materials & methods:**

Commercially available products were used to develop a novel, low-cost model for ultrasound-guided aspiration and I&D of an abscess. A latex balloon embedded in silicone gel construct simulated a superficial fluid collection when examined with an ultrasound probe and monitor. A 18-gauge needle on a 10-cc syringe were used for aspiration, and a 15-blade disposal scalpel with 0.25″ packing strip used for I&D.

**Results:**

Approximately six hours are required to generate 24 individual models of a superficial abscess. Following an initial investment, each model costs less than $1 USD to produce. Compared to commercially available models, this represents a significant savings. This model was utilized during the medical school academic year as a teaching aid for medical students to simulate ultrasound-guided identification, aspiration, and incision and drainage of a superficial abscess.

**Conclusions:**

We successfully produced an affordable, low-cost model of a superficial fluid collection for training in ultrasound usage, aspiration, and I&D. The model represents significant savings over commercially available alternatives and can be easily replicated for trainee education.

## Introduction

Superficial skin and soft-tissue abscesses are a common chief complaint in the emergency department, with up to 3.2 % of patients presenting with this issue [1]. Commonly identified with ultrasound guidance, the preferred method for treatment is incision and drainage (I&D) as antibiotics alone are often insufficient. As a result, incision and drainage of a superficial or perianal abscess is an important skill for a range of providers in the primary care, urgent care, emergency rooms, and general surgery fields [2,3]. Fittingly, the Surgical Council on Resident Education (SCORE) has identified it as a core concept in surgical education [4]. Finally, the effective use of ultrasound is becoming increasingly important to surgical education, improving the safety of many bedside and operating room procedures. To this end, the National Ultrasound Faculty of the American College of Surgeons was developed to develop and implement a curriculum and certification in ultrasound use and applications [[5], [6], [7]].

Despite the prevalence of superficial abscesses in the U.S. population and the role of general surgery in their treatment, there is little education on the appropriate technique to perform this procedure in medical school. Unfortunately, few commercially available models exist to practice and teach this concept and the models are often cost-prohibitive. Furthermore, it is unlikely these commercials models would be affordable for global surgical education efforts. Additionally, although physical exam is often an appropriate diagnostic tool to identify abscess location, ultrasound imaging can be useful in determining the complexity and size of the abscess, as well as identification of appropriate incision sites for effective drainage [8,9]. To provide an affordable option for trainee education and simulation, we sought to develop a low-cost model of a superficial abscess for education in ultrasound use and identification, aspiration, and I&D.

## Materials and methods

### Abscess model generation

A complete step-by-step guide with images is provided in Supplementary Material 1 and a complete list of supplies with approximate costs is in Table 1. Briefly, sixteen ounces by volume of Ecoflex™ 00–30 Silicone Rubber Part B (Smooth-on Inc., Macungie, PA) is measured and added to a measuring cup with one mL of flesh-tone Silcpig™ pigment (Smooth-on Inc., Macungie, PA). Sixteen ounces by volume of Ecoflex™ 00–30 Silicone Rubber Part A (Smooth-on Inc., Macungie, PA) is measured and combined into the same measuring cup. One tbsp of psyllium powder is added, and the mixture is stirred for two minutes. The silicone mixture is then placed into a 24-count muffin tray until approximately 2/3 full and incubated at room temperature for two hours, until the silicone is partially set. A series of Squiggly Latex Balloons (Amscan Manufacturing, Milton Keynes, United Kingdom) are filled with tap water, each individual balloon is isolated via tying off the ends, and the individual balloon is placed in the center of the model.

The top of the abscess model and balloon are covered with an additional silicone layer. Eight ounces by volume of Ecoflex™ 00–30 Silicone Rubber Part B is measured and combined with 0.5 mL of flesh-tone Silcpig™ pigment. This mixture is added to eight ounces by volume of Ecoflex™ 00–30 Silicone Rubber Part A and 1 teaspoon of psyllium powder, then stirred for two minutes. The final blend is then poured over the models until they are completely covered, allowed to incubate at room temperature for 2–4 h, and removed from the tray for storage.

**Table 1:** Approximate costs of the necessary reagents to generate the model as described in the Materials and Methods.

Material	Manufacturer	Approximate Sales Cost
EcoFlex 00–30 Silicone Rubber Part A and B	Smooth-On (Trial size, 2 lbs)	32.90
	Smooth-on (1 gal, 16 lbs)	215.99
	Smooth-on (55 gal, 880 lbs)	Not stated
SilcPig Silicone Pigment	Smooth-On (9 pack)	31.80
Measuring Cup	Norpro 3035	2.49
Paint Stirrer	TCP Global Wood (50 pack)	6.99
Psyllium powder	Now Foods (24 oz. canister)	28.95
Squiggly Latex Balloons	Unique Store (15 balloons)	7.02
24 count muffin tin	Wilton Baker's Best	21.88

### Model testing and use

An ultrasound probe was used to assess the consistency and reproducibility of the model from various angles. The models were tested with a 16-, 18-, 22-, and 25-gauge needle (McKesson Medical-Surgical, Jacksonville, FL, USA) to determine the appropriate size for trainee education and use. To test the models' potential to teach I&D, a 15-blade scalpel (Aspen Surgical, Caledonia, MI, USA) was used to make a cruciate incision in the tissue and latex balloon, and forceps used to place a 0.25″ x 5 yard packing strip (Curity, Covidien, Minneapolis, MN) in the wound.

## Results

### Abscess model generation

The described recipe to generate 24 individual abscess models would cost in total approximately $132 USD ($5.50 USD a model). The most expensive material is the Ecoflex™ 00–30 Silicone Rubber, with a cost that can be decreased significantly if ordered in bulk up to 880 pounds (compared to the trial size of two pounds). The intermediate consumer price point is the one-gallon (16 pound) version which reduces the cost of each individual model to approximately $0.88 USD after the initial purchase of reusable products like paint stirrer, balloons, and the measuring tins. This is considerably cheaper than the commercially available products (Table 2).

**Table 2:** Alternative abscess models and associated sales price of the model (not including shipping).

Model	Manufacturer	SKU	Approximate Sales Price
Abscess Drainage Ultrasound Training Model	Blue Phantom, CAE Healthcare, Sarasota, Fl	A-108337	1122.08
Ultrasound Deep Abscess Trainer	Your Design Medical, New York, NY	Not stated	199.99

**Fig. 1:**
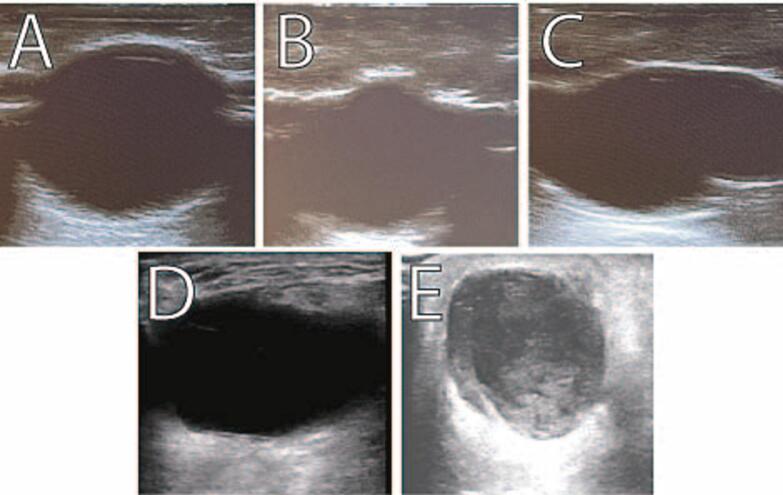
Representative examples of the abscess model (A, B, C) as seen on ultrasound. The images in D and E represent a real-life ultrasound evaluation of an abscess [10,11].

The models demonstrate significant consistency and reproducibility, with slight variations in size or depth of the fluid collection depending on placement of the latex balloon (Fig. 1A-C). The models compare favorably to images taken from an internet search for abscess fluid collections (Fig. 1D,E) [10,11]. Additionally, a small quantity of psyllium powder can be added to the latex balloon to simulate a complex fluid collection and component layering. The models tested with needle diameter larger than and including a 22-gauge needle were successfully able to aspirate the fluid collection.

### Trainee education

The described model was used to teach medical students on the general surgery clerkship the steps of ultrasound usage, aspiration, and I&D throughout the 2022–2023 academic year (approximately 100 students). The students were instructed how to appropriately orient the ultrasound probe, recognize a fluid collection and the differences between tissue densities, hold a syringe, and use a syringe with a needle to enter the tissue and aspirate - while tracking the needle with the probe. The students were then instructed how to appropriately perform a cruciate incision for I&D, remove sufficient tissue to prevent premature wound closure, and place packing strip in the wound using forceps. A discussion was held on the indications for, risks and benefits, and required consent for each procedure.

## Discussion

Although it is a common consult for general surgery residents throughout the country, there are few educational models to train residents and other learners on superficial abscess care. Our developed model is significantly cheaper than the closest commercial competitor ($0.88 compared to over $30 USD), reproducible, customizable, and easily constructed with simple reagents. The two commercially available models found online vary between ∼$200–1100 in cost. It is unclear why these models are so expensive; the reported cost is solely the sales price and excludes shipping and handling charges. While we do not have access to the commercial models, it is possible the models are more complex or durable than the described model, though they do not appear to be significantly complex based on the product description. The increased cost may also come from a higher overhead associated with creating, marketing, and storing, and selling the product. Our experience with our model suggests them to be durable, reproducible for aspiration and drainage education, and much cheaper than the alternative.

A significant limitation of this study is the lack of data regarding the model's use in an educational curriculum. We initially developed and used this model for medical student education, though we have since included it in our general surgery intern orientation and “bootcamp” for residency. Verbal feedback from medical students and residents is overwhelmingly positive. Formal studies are planned regarding the effect of implementing this model in medical student surgical clerkship and surgical intern orientation.

Ultrasound is becoming an increasingly common adjunct to the physical exam in the hospital setting. There are many benefits to incorporating ultrasound into daily practice; as the technology progresses to decrease the size and improve the portability, it will likely become a ubiquitous tool. As such, developing models, such as the one described, to improve the trainee education with ultrasound will be of importance in the coming years. Though initially designed for I&D education, the anecdotal experience of medical students and residents suggests there are other potential applications. For example, this model could potentially be adapted to replicate the Seldinger technique of vascular access for central or arterial lines. Longer models equipped with longer latex balloons could serve as “vessels” to teach trainees how to use an ultrasound to track a needle to gain vascular access, thread a wire, remove the needle, dilate the vessels, and place a central or arterial line.

In conclusion, this report describes the development of a novel, low-cost model for superficial abscess ultrasound identification and treatment. The model is of value in trainee education with regards to ultrasound usage, abscess identification, aspiration, and I&D. The low price-point and ease of construction decrease the financial barriers that may be present to this educational opportunity.

The following are the supplementary data related to this article.

Supplementary material 1 A step-by-step detailed description of how to generate the superficial abscess model. Supplementary material 1

Supplementary video A video depiction of the abscess model in use, including aspiration and incision & drainage. Supplementary video

## CRediT authorship contribution statement

Conception and Design – JML, LK, LT, BF, CJV

Analysis and Interpretation – JML, LK, BF, LT, CJV

Data Collection – JML, LK, LT, BF, CJV

Writing the article – JML, LK, JM, SZ, LJ, CJV

Critical Revision of manuscript –JML, SZ, LJ, CJV

## Ethical approval

This proposal was determined to be exempt from IRB review.

## Funding

JML is a recipient of the Duke Resident Physician-Scientist Program NIAID R38 (1R38AI140297).

## Declaration of competing interest

The authors report no conflicts of interest or financial disclosures.

## References

[1] Hersh A.L., Chambers H.F., Maselli J.H., Gonzales R. (2008). National trends in ambulatory visits and antibiotic prescribing for skin and soft-tissue infections. Arch Intern Med.

[2] Hauk L. (2018). Antibiotics for uncomplicated skin abscesses after incision and drainage: BMJ rapid recommendation. afp.

[3] Vermandere M. (2018). Antibiotics after incision and drainage for uncomplicated skin abscesses: a clinical practice guideline. BMJ.

[4] SCORE Curriculum Outline | American Board of Surgery https://www.absurgery.org/default.jsp?scre_booklet.

[5] Nassour I., Spalding M.C., Hynan L.S., Gardner A.K., Williams B.H. (2017). The surgeon-performed ultrasound: a curriculum to improve residents’ basic ultrasound knowledge. J Surg Res.

[6] Staren E.D. (2006). An evaluation of the American College of Surgeons’ ultrasound education program. Am J Surg.

[7] Knudson M.M., Sisley A.C. (2000). Training residents using simulation technology: experience with ultrasound for trauma. J Trauma Acute Care Surg.

[8] Blaivas M., Adhikari S. (2011). Unexpected findings on point-of-care superficial ultrasound imaging before incision and drainage. J Ultrasound Med.

[9] Squire B.T., Fox J.C., Anderson C. (2005). ABSCESS: applied bedside sonography for convenient evaluation of superficial soft tissue infections. Acad Emerg Med.

[10] (2020). Skin and Soft Tissue Infections: A PoCUS Guided Approach | Department of Emergency Medicine | Saint John. https://sjrhem.ca/skin-and-soft-tissue-infections-a-pocus-guided-approach/.

[11] Abscess Evaluation. https://www.acep.org/sonoguide/procedures/abscess-evaluation.

