# Supplementary material for: A novel low-cost model of superficial abscess for trainee education in incision and drainage
Source: Surg Open Sci. 2023 Jul 27;14:124–7. doi: 10.1016/j.sopen.2023.07.015 (PMC10428102; doi:10.1016/j.sopen.2023.07.015)

**SUPPLEMENTARY MATERIAL**


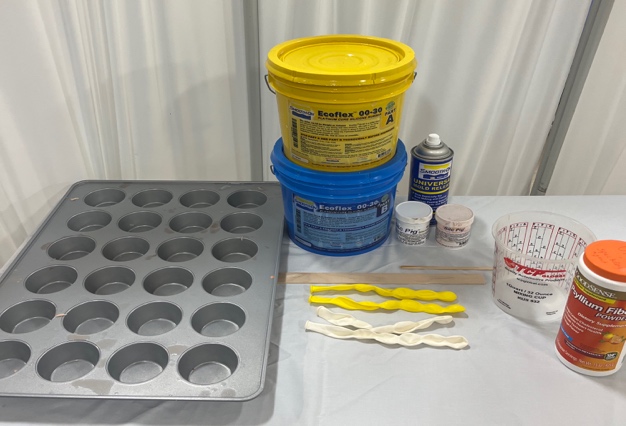


1. Start by pouring 16 oz. of Ecoflex part B into the measuring cup and add a dab of flesh tone Silcpig pigment.


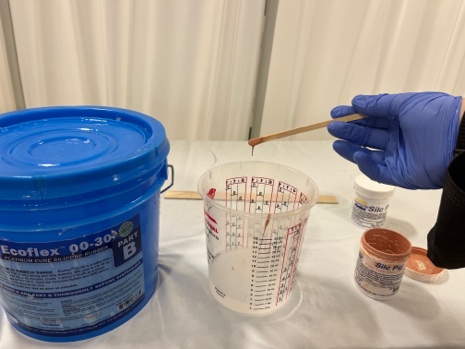

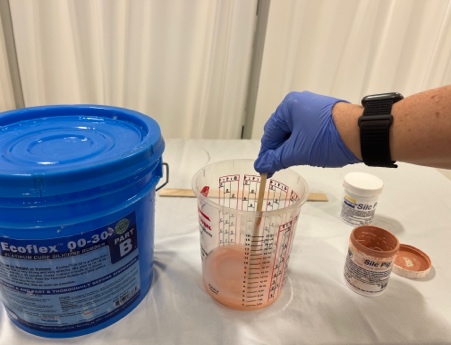


1. Then pour 16 oz. of Ecoflex Part A into the same measuring cup, add 1 tbsn of Psyllium powder and mix with paint stirrer for 2 minutes.


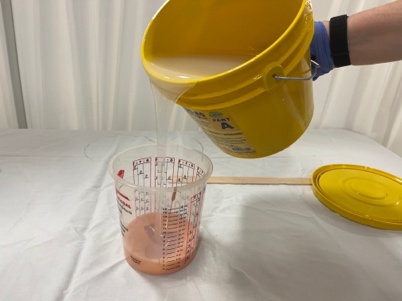

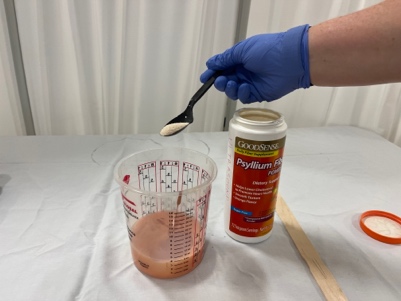

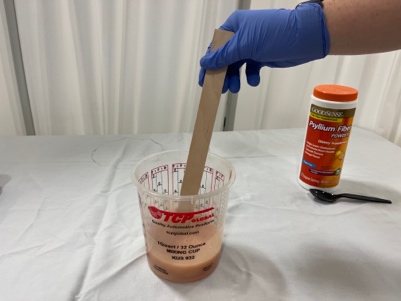


1. Once mixed, pour silicone into muffin cups until 2/3 full.


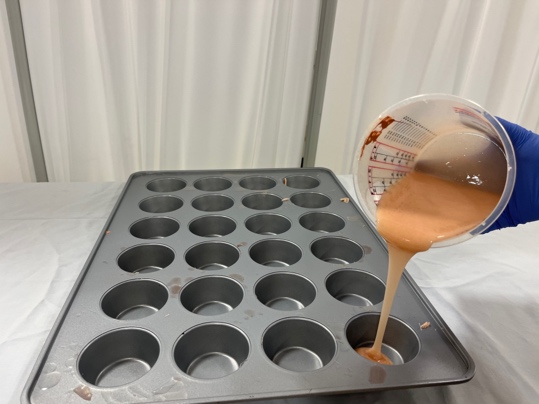


1. Wait 2 hours until silicone is almost set.
2. While waiting fill small water balloons with a few milliliters of water.


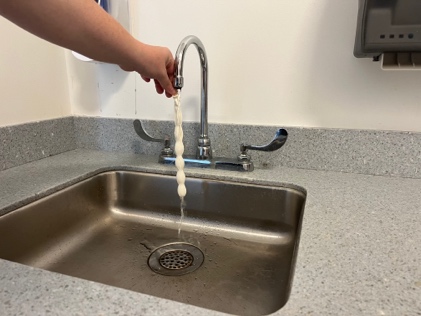

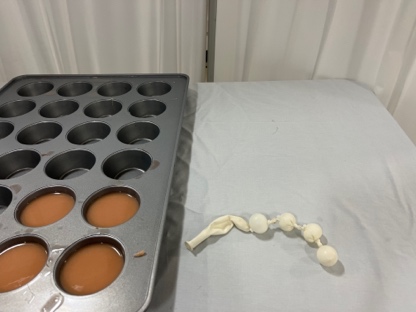

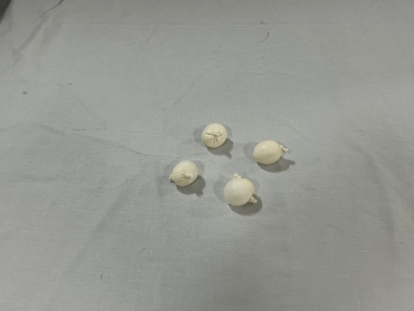


1. Before silicone is completely set place the water balloons in the center of each model.


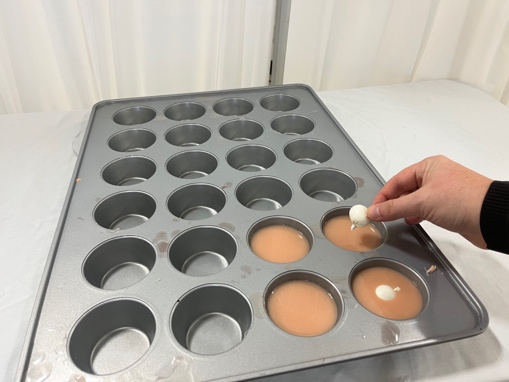

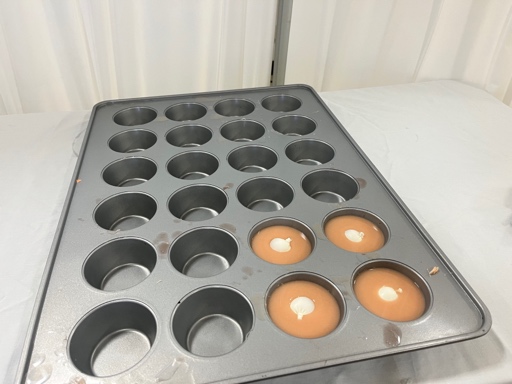


1. Pour 8oz. of Ecoflex part B into the measuring cup and add a dab of flesh tone Silcpig pigment to match what was already poured.


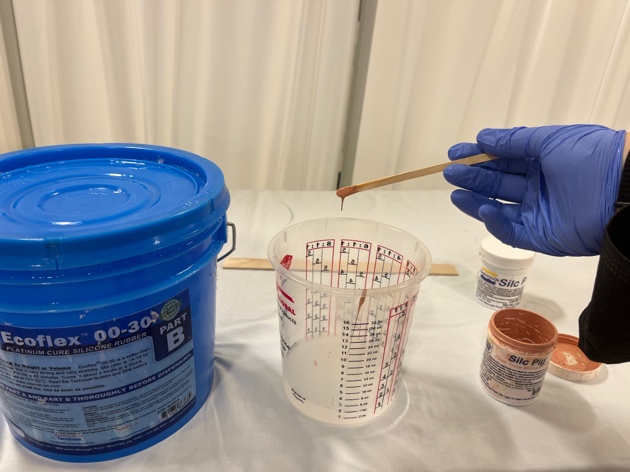

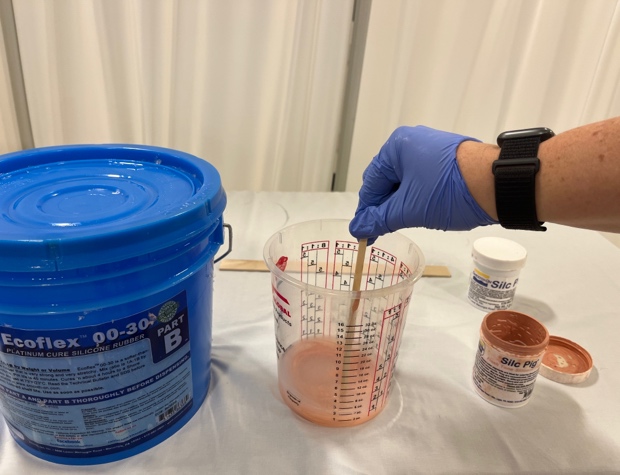


1. Then pour 8 oz. of Ecoflex Part A into the same measuring cup, add 1 tsp of Psyllium powder and mix with paint stirrer for 2 minutes.


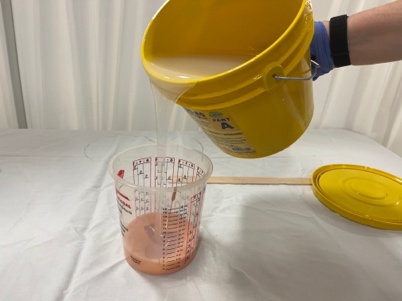

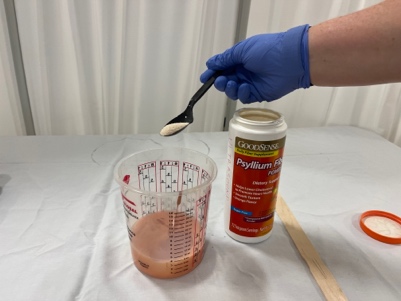

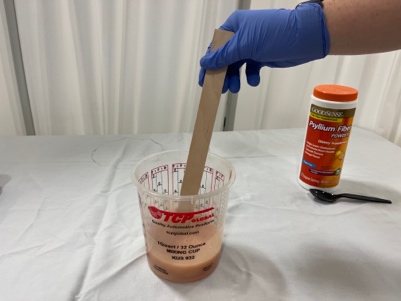


1. Pour over the balloons until they are completely covered.


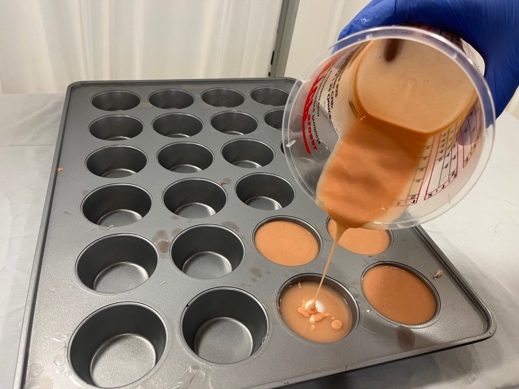

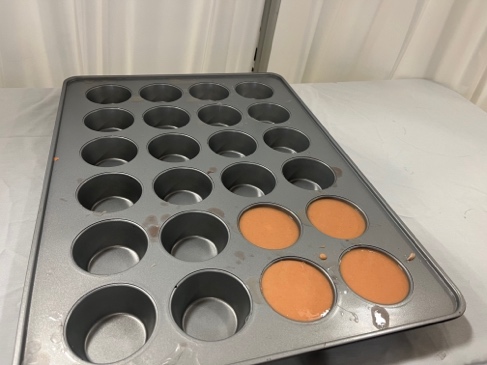


1. Let sit for 2 to 4 hours until the silicone has completely set.


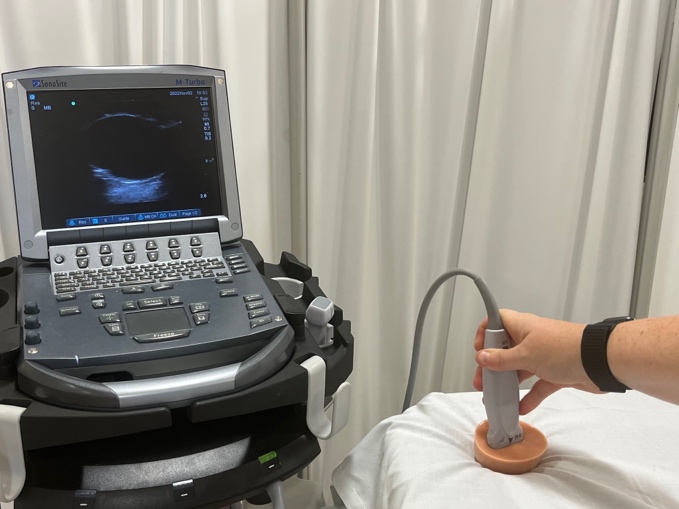

Supplement: Supplementary material 1 — A step-by-step detailed description of how to generate the superficial abscess model. [file mmc1.docx]
